# *De novo* transcriptome assemblies of four xylem sap-feeding insects

**DOI:** 10.1093/gigascience/giw007

**Published:** 2017-02-24

**Authors:** Erica E. Tassone, Charles C. Cowden, S.J. Castle

**Affiliations:** 1 Plant Physiology and Genetics Research Unit, U.S. Arid Land Agricultural Research Center, USDA ARS, Maricopa, AZ USA; 2 Pest Management and Biocontrol Research Unit, U.S. Arid Land Agricultural Research Center, USDA ARS, Maricopa, AZ USA

**Keywords:** Transcriptome, RNA-seq, Trinity, Insect herbivory, Insect vector, Diet specialization

## Abstract

**Background:** Spittle bugs and sharpshooters are well-known xylem sap-feeding insects and vectors of the phytopathogenic bacterium *Xylella fastidiosa* (Wells), a causal agent of Pierce's disease of grapevines and other crop diseases. Specialized feeding on nutrient-deficient xylem sap is relatively rare among insect herbivores, and only limited genomic and transcriptomic information has been generated for xylem-sap feeders. To develop a more comprehensive understanding of biochemical adaptations and symbiotic relationships that support survival on a nutritionally austere dietary source, transcriptome assemblies for three sharpshooter species and one spittlebug species were produced.

**Findings:** Trinity-based *de novo* transcriptome assemblies were generated for all four xylem-sap feeders using raw sequencing data originating from whole-insect preps. Total transcripts for each species ranged from 91 384 for *Cuerna arida* to 106 998 for *Homalodisca liturata* with transcript totals for *Graphocephala atropunctata* and the spittlebug *Clastoptera arizonana* falling in between. The percentage of transcripts comprising complete open reading frames ranged from 60% for *H. liturata* to 82% for *C. arizonana*. Bench-marking universal single-copy orthologs analyses for each dataset indicated quality assemblies and a high degree of completeness for all four species.

**Conclusions:** These four transcriptomes represent a significant expansion of data for insect herbivores that feed exclusively on xylem sap, a nutritionally deficient dietary source relative to other plant tissues and fluids. Comparison of transcriptome data with insect herbivores that utilize other dietary sources may illuminate fundamental differences in the biochemistry of dietary specialization.

## Data description

### Background

Resource partitioning among herbivorous insects spans a continuum between specialists that feed on one or a few plant species to generalists that are able to utilize hundreds of species belonging to multiple plant families. A further element of plant partitioning involves the particular location on a plant or tissue type from which an insect feeds [1]. The diversity of plant feeding strategies has evolved along with specialized anatomical features such as mouthparts and digestive systems, unique enzyme complements for processing plant compounds, and partnerships with symbiotic microbiota that contribute to nutritional gain of the host insect. The transcriptome assemblies presented here include four species that feed exclusively on sap from xylem vessels, a relatively rare form of plant feeding from a source that among plant tissues is the most deficient in nitrogen and carbon content [2]. Three of the transcriptomes represent sharpshooter species that are members of the subfamily Cicadellinae (Cicadellidae) that belong to the superfamily Membracoidea (leafhoppers, sharpshooters, treehoppers). The fourth transcriptome represents a spittlebug (Clastopteridae) that belongs to the superfamily Cercopoidea (spittlebugs, froghoppers). All are members of the hemipteran suborder Auchenorrhyncha [3]. Their piercing-sucking mouthparts tap into xylem vessels from which sap is consumed in copious quantities to compensate for its low nutritional value. Sharpshooters are recognized for their efficient assimilation of limited nutrients in xylem sap [4], but putative biochemical mechanisms that enable specialization on xylem sap are unknown. Also unclear is whether the respective roles in host nutrition played by the dual primary endosymbionts are consistent among xylem feeders [5]. Comparison of transcriptomes of four xylem-feeding insects will provide additional knowledge and insight into the survival of ecological specialists on a nutritionally impoverished dietary source.

### Samples

The spittlebug *Clastoptera arizonana* Doering was collected in 2014 from a wild population infesting grapevines in Yavapai County, AZ and established as a glasshouse colony for 8 months prior to sample collection in Maricopa, AZ. Samples of the sharpshooter *Cuerna arida* Oman and Beamer (tribe Proconiini) were collected in 2015 by sweep net from a wild population in mixed vegetation in Cochise County, AZ. The smoke-tree sharpshooter *Homalodisca liturata* Ball (Proconiini) was collected in 2015 from *Euphorbia tirucalli* L. plants in Phoenix, AZ. The blue-green sharpshooter *Graphocephala atropunctata* (Signoret) (Cicadellini) was collected in 2013 from a wild population in Orange County, CA and maintained as a glasshouse colony on basil (*Osimium basilicum* L.) until samples were collected in 2015. Live adults of unknown age from all four species were homogenized separately in RNA*later* (Ambion/Life Technologies, Carlsbad, CA) and stored at −20°C. Total RNA extractions were performed using a Qiagen RNeasy mini kit followed by quality assessment on an Agilent 2100 bioanalyzer. Library generation yielding 2 × 100 bp paired-end reads (TruSeq RNA Sample Preparation Kit v2; Illumina Inc., San Diego, CA) and sequencing (Illumina HiSeq2000 or HiSeq2500) were performed at the University of Arizona Genomics Center in Tucson, AZ (http://uagc.arl.arizona.edu).

## Data filtering

The total number of reads, data quantity, and short read archive numbers for each of the four xylem-feeding insects are shown in Table 1. For each data set, raw quality was assessed and filtered using both FastQC and Trimmomatic (v 0.32) using the parameters ILLUMINACLIP:TruSeq3-PE.fa:2:30:10 LEADING:10 TRAILING:20 SLIDINGWINDOW:4:25 MINLEN:36 to remove adaptor sequence and filter by quality score.

**Table 1.** Accession numbers for sequence reads and assembled transcripts for four species of xylem-feeding insects

Sample	Reads	Size (Gb)	Short Read Archive	BioSample	BioProject
*Homalodisca liturata*	18 936 520	18.9	SRX1451710	SAMN04293489	PRJNA303151
			SRX1451711	SAMN04293490	“
			SRX1451712	SAMN04293491	“
*Clastoptera arizonana*	19 038 998	17.8	SRX1451715	SAMN04293493	PRJNA303152
			SRX1451717	SAMN04293494	“
			SRX1451718	SAMN04293495	“
*Cuerna arida*	14 667 040	18.3	SRX1451216	SAMN04292971	PRJNA303150
			SRX1451218	SAMN04292972	“
			SRX1451467	SAMN04292973	“
*Graphocephala atropunctata*	16 868 134	8.2	SRX1411425	SAMN04208332	PRJNA299492
			SRX1411426	SAMN04208333	“
			SRX1411427	SAMN04208334	“

## Transcriptome assembly

All raw data for each insect transcriptome were run through the following pipeline. Prior to assembly, the three replicate samples were concatenated and read abundance was normalized to 50× coverage using the *in silico* normalization tool in Trinity v. 2.0.6 [6] to improve assembly time. Each of the datasets was assembled in Trinity using the default parameters, with the addition of the ‘–jaccard clip’ flag to reduce the generation of transcript fusions from non-strand specific data. Open reading frames were predicted using Transdecoder with all run parameters set to default [6]. The transcriptomes were filtered, sorted, and prepared for NCBI transcriptome shotgun assembly (TSA) submission as previously described [7]. To comply with NCBI TSA submission, all transcripts resulting from endosymbionts or bacteria were removed from the final assembly prior to submission.

## Annotation

Functional annotation for each of the transcriptomes was performed at the peptide level using a custom pipeline [7] that defines protein products and assigns transcript names. Predicted proteins and peptides were analyzed using InterProScan 5 [8], using the ‘–iprlookup’ and ‘–goterms’ flags, to search all available databases, including Gene Ontology. Each transcriptome was annotated using BLASTp against the UniProt Swiss Prot database (downloaded 11 February 2015). Annie [9], a program that cross-references SwissProt BLAST and InterProScan5 results to extract qualified gene names and products, was used to generate the transcript annotation file. The resulting .gff3 and .tbl files were further annotated with functional descriptors in Transvestigator [10].

## Transcriptome Quality and Comparisons

Assembled transcriptome metrics showed a high percentage of reads mapping back to each transcriptome (Table 2) indicating successful assemblies. TransRate [11] scores ranging from 0.16 to 0.42 were used for quality assessment, and bench-marking universal single-copy orthologs (BUSCO) v. 1.1.b1 results using the arthropod gene set (downloaded December 19, 2015) [12] indicated that the four transcriptomes have a moderate to high level of completeness. It should be noted that both the TransRate value (0.16) and BUSCO results for *H. liturata* suggest this transcriptome may contain more partial transcripts than the other three assemblies.

**Table 2.** Transcriptome assembly statistics and results of BUSCO analysis for four xylem-feeding insects

	*H. liturata*	*C. arizonana*	*C. arida*	*G. atropunctata*
Assembly				
Normalized reads	9 468 260	9 519 499	10 714 375	32 429 458
Total no. transcripts	106 998	93 845	91 384	97 830
Average transcript length and range	954 (224–30 062)	1232 (224–29 936)	901 (224–20 095)	962 (224–17 082)
Total assembled bases (all)	102 317 189	115 686 868	79 785 471	94 141 447
N50 (all)	1650	2510	1560	1692
% GC	37	31	37	39
% mapping	84	91	88	95
TransRate Score	0.16	0.28	0.25	0.42
BUSCO				
Complete (%)	60	82	68	66
Duplicated (%)	23	42	26	24
Fragmented (%)	23	9.2	17	19
Missing (%)	15	8	14	13

Each of the assembled transcriptomes was used in a reciprocal tBLASTx search to identify similarities between the four species and their transcriptome assemblies. The final, filtered transcriptomes were made into nucleotide BLAST databases using NCBI Blast+ (v 2.2.30) *makeblastdb* tool, and all tBLASTx searches were performed using an e-value cutoff of 1e^−3^_._ The tBLASTx results (Table 3) indicate similarities between the four xylem-feeder transcriptomes, with the lowest (38%) occurring between members of the two superfamilies (spittlebug and all sharpshooters) and the highest (84%) between *H. liturata* and *C. arida*, members of the same subfamily [13].

**Table 3.** Total percent matches from tBLASTx reciprocal searches. Transcriptome used as query on the left, and nucleotide database tBLASTx against which the query was performed is shown at the top

	Nucleotide Database (% similarity)			
	*H. liturata*	*C. arizonana*	*C. arida*	*G. atropunctata*
Query				
*Homalodisca liturata*	–	42.94	76.31	56.20
*Clastoptera arizonana*	40.90	–	38.35	38.58
*Cuerna arida*	83.90	43.40	–	58.36
*Graphocephala atropunctata*	56.86	40.30	56.10	–

e-value ≤ 1^E-3^

## Availability of supporting data

The filtered and annotated transcriptomes have been deposited in GenBank as a TSA under the accessions and BioProject numbers found in Table 1. Datasets further supporting the results of this article are available in the *GigaScience* repository, GigaDB [13].

### Abbreviations

BUSCO: bench-marking universal single-copy orthologs; TSA: transcriptome shotgun assembly; USDA-ARS: United States Department of Agriculture-Agricultural Research Service

### Competing Interests

The authors declare that they have no competing interests.

### Authors' Contribution

SJC and CCC conceived and performed the experiments; EET analyzed the data and evaluated the conclusions; EET, SJC, and CCC wrote the manuscript. All authors approved the final manuscript.

## Supplementary Material

GIGA-D-16-00077_Original-Submission.pdf

GIGA-D-16-00077_Revision_1.pdf

Response_to_Reviewer_Comments_Original_Submission.pdf

Reviewer_1_Report_(Original_Submission).pdf

Reviewer_2_Report_(Original_Submission).pdf

Reviewer_3_Report_(Original_Submission).pdf
