# Supplementary material for: De novo transcriptome assemblies of four xylem sap-feeding insects
Source: Gigascience. 2017 Feb 24;6(3):1–4. doi: 10.1093/gigascience/giw007 (PMC5467018; doi:10.1093/gigascience/giw007)
Supplement: Response_to_Reviewer_Comments_Original_Submission.pdf [file giw007_Response_to_Reviewer_Comments_Original_Submission.pdf]

Reviewer #1
Response: New text addressing insect stage and age, RNA extraction, and library preps was added to the Samples section lines 57-61.

Reviewer #2
Response: New text was added to the Transcriptome Assembly section indicating that all endosymbiont or bacterial transcripts were removed prior to submission to NCBI (lines 78-82).

Reviewer #3
Page 4 line 90
Response: As indicated to Reviewer 2, bacterial sequences were removed from the assembly. Given the brevity of the Data Note format, we decided to withhold the endosymbiont data to use potentially in a follow-up report. Thus, for the present manuscript, no comment on the endosymbiont reads beyond what was added seems appropriate.

Also – NCBI does not allow you to submit the dataset with the endosymbiont data intact, to be approved for a TSA, they require these reads be removed.

Page 5 line 106
Response: New text added explaining that transdecoder was set to default (lines 78-79).

Response to Reviewer 3 question about G. atropunctata: We can only speculate on why this is the case, e.g. perhaps different lanes of the sequencer flowcell were more efficient yielding data because they were less contaminated from prior samples and were able to yield more data.

Response to Reviewer 3 question about transrate scores: The transrate scores for all four species were used only to assess the assembly, and the file generated from the program was not used as part of the final assembly. The scores are referred to in the Annotation section and included in a Table 2 row with the heading “TransRate Score”.
