# Supplementary material for: De novo transcriptome assemblies of four xylem sap-feeding insects
Source: Gigascience. 2017 Feb 24;6(3):1–4. doi: 10.1093/gigascience/giw007 (PMC5467018; doi:10.1093/gigascience/giw007)
Supplement: Reviewer_1_Report_(Original_Submission).pdf [file giw007_Reviewer_1_Report_(Original_Submission).pdf]

Reviewer Report

Title: "De novo transcriptome assemblies of four xylem sap-feeding insects"

Version: Original Submission **Date:** 9/16/2016

Reviewer name: Rodolphe Poupardin

Reviewer Comments to Author:

In the present Data note, the authors submitted de-novo transcriptome assemblies for four different xylem-sap feeding insects (H liturata, C arizonana, C arida, G atropruncta): Because of the particular feeding behaviour of these insects that feed exclusively on xylem sap, these datasets clearly improve the herbivory collection of insects belonging to this niche. Moreover, it will a valuable source of data for people interested in comparing how insects can cope and adapt to food with low nutritional value. The pipeline authors used is adequate. These methods have been intensively tested and published. I have checked all access numbers and it seems that authors correctly uploaded all their RAW and processed files to NCBI and GigaScience databases. The RAW reads are not yet publicly accessible but I am sure they will become after acceptance of the manuscript. I believe this data note is suitable for publication in GigaScience journal with only few minor revisions. I would like the authors to provide a little bit more information in the M&M part:how old were the insects when frozen? …. (some transcripts are differentially regulated depending on the life stage and age, and thus may not be detected) which RNA extraction kit did they use?Can authors give a little bit more details about the library preparations?Read size? paired-end I presume?These are minor comments, but I think it is important for anyone interested in using these datasets .

Level of Interest

Please indicate how interesting you found the manuscript: An article whose findings are important to those with closely related research interests

Quality of Written English

Please indicate the quality of language in the manuscript: Acceptable

Declaration of Competing Interests

Please complete a declaration of competing interests, considering the following questions:

- Have you in the past five years received reimbursements, fees, funding, or salary from an organisation that may in any way gain or lose financially from the publication of this manuscript, either now or in the future?
- Do you hold any stocks or shares in an organisation that may in any way gain or lose financially from the publication of this manuscript, either now or in the future?

- Do you hold or are you currently applying for any patents relating to the content of the manuscript?
- Have you received reimbursements, fees, funding, or salary from an organization that holds or has applied for patents relating to the content of the manuscript?
- Do you have any other financial competing interests?
- Do you have any non-financial competing interests in relation to this paper?

If you can answer no to all of the above, write 'I declare that I have no competing interests' below. If your reply is yes to any, please give details below.

I declare that I have no competing interests

I agree to the open peer review policy of the journal. I understand that my name will be included on my report to the authors and, if the manuscript is accepted for publication, my named report including any attachments I upload will be posted on the website along with the authors' responses. I agree for my report to be made available under an Open Access Creative Commons CC-BY license (http://creativecommons.org/licenses/by/4.0/). I understand that any comments which I do not wish to be included in my named report can be included as confidential comments to the editors, which will not be published.

I agree to the open peer review policy of the journal
