# Supplementary material for: De novo transcriptome assemblies of four xylem sap-feeding insects
Source: Gigascience. 2017 Feb 24;6(3):1–4. doi: 10.1093/gigascience/giw007 (PMC5467018; doi:10.1093/gigascience/giw007)
Supplement: Reviewer_2_Report_(Original_Submission).pdf [file giw007_Reviewer_2_Report_(Original_Submission).pdf]

Reviewer Report

Title: "De novo transcriptome assemblies of four xylem sap-feeding insects"

Version: Original Submission **Date:** 9/23/2016

Reviewer name: Peter Ashton, PhD

Reviewer Comments to Author:

This is a simple data note, which describes the collection, assembly and deposition of RNA transcript data from four important species of sap feeding insects. The methods used to prepare the data for dissemination are entirely appropriate and the data will be very useful to those who are working in this field. I have no issue with the paper being published as it stands.Having said that, I would probably suggest that the authors consider the possibility that some of the sequences in their finished transcriptomes might well come from the endosymbionts which at least some of these species host. It would be nice (as someone who has worked on endosymbionts) to see this at least mentioned in this manuscript, specially since there are now so many of these endosymbionts already fully sequenced and in the NCBI database. However, if the authors do not see this as being appropriate, I would not suggest that this should hold up publication of the manuscript.

Level of Interest

Please indicate how interesting you found the manuscript: An article whose findings are important to those with closely related research interests

Quality of Written English

Please indicate the quality of language in the manuscript: Acceptable

Declaration of Competing Interests

Please complete a declaration of competing interests, considering the following questions:

- Have you in the past five years received reimbursements, fees, funding, or salary from an organisation that may in any way gain or lose financially from the publication of this manuscript, either now or in the future?
- Do you hold any stocks or shares in an organisation that may in any way gain or lose financially from the publication of this manuscript, either now or in the future?
- Do you hold or are you currently applying for any patents relating to the content of the manuscript?
- Have you received reimbursements, fees, funding, or salary from an organization that holds or has applied for patents relating to the content of the manuscript?
- Do you have any other financial competing interests?

- Do you have any non-financial competing interests in relation to this paper?

If you can answer no to all of the above, write 'I declare that I have no competing interests' below. If your reply is yes to any, please give details below.

1. No 2. No 3. No 4. No 5. No 6. No

I agree to the open peer review policy of the journal. I understand that my name will be included on my report to the authors and, if the manuscript is accepted for publication, my named report including any attachments I upload will be posted on the website along with the authors' responses. I agree for my report to be made available under an Open Access Creative Commons CC-BY license (http://creativecommons.org/licenses/by/4.0/). I understand that any comments which I do not wish to be included in my named report can be included as confidential comments to the editors, which will not be published.

I agree to the open peer review policy of the journal
