# Supplementary material for: De novo transcriptome assemblies of four xylem sap-feeding insects
Source: Gigascience. 2017 Feb 24;6(3):1–4. doi: 10.1093/gigascience/giw007 (PMC5467018; doi:10.1093/gigascience/giw007)
Supplement: Reviewer_3_Report_(Original_Submission).pdf [file giw007_Reviewer_3_Report_(Original_Submission).pdf]

Reviewer Report

Title: "De novo transcriptome assemblies of four xylem sap-feeding insects"

Version: Original Submission **Date:** 10/4/2016

Reviewer name: Sebastian Eves-van den Akke

Reviewer Comments to Author:

I recommend that manuscript GIGA-D-16-00077 entitled, "De novo transcriptome assemblies of four xylem sap-feeding insects" is accepted for publication following minor revisions.All the data associated with the submission appear to be available on the FTP, and the raw data associated with the manuscript is apparently uploaded to the SRA, although not yet available for inspection. The approaches seem technically sound, and I have only minor comments - page and line numbers refer to those in the pdf.Minor comments:Page 4 line 90.Am I correct in reading that total RNA was sequenced, or was poly A pull down part of the library preparation step. Regardless, should the authors comment on the assembly of endosymbiont reads within the dataset where appropriate?Page 5 line 106:Could the settings used for transdecoder be provided (whether or not PFAM domain information was included and what the CDHIT threshold was set to if not default).Given that all libraries were normalised to 50x coverage before assembly, is it necessary to comment on why the number of reads that assembled in G. actropunctata transcripts is much higher than the other species, while the size of the assembly (by any metric provided in table 2) is not?To be included in the methods, was transrate used to triage the transcripts (and thus help give rise to the "final" assembly, or simply asses their quality. If the later, can these data also be provided?

Level of Interest

Please indicate how interesting you found the manuscript: An article of importance in its field

Quality of Written English

Please indicate the quality of language in the manuscript: Acceptable

Declaration of Competing Interests

Please complete a declaration of competing interests, considering the following questions:

- Have you in the past five years received reimbursements, fees, funding, or salary from an organisation that may in any way gain or lose financially from the publication of this manuscript, either now or in the future?
- Do you hold any stocks or shares in an organisation that may in any way gain or lose financially from the publication of this manuscript, either now or in the future?

- Do you hold or are you currently applying for any patents relating to the content of the manuscript?
- Have you received reimbursements, fees, funding, or salary from an organization that holds or has applied for patents relating to the content of the manuscript?
- Do you have any other financial competing interests?
- Do you have any non-financial competing interests in relation to this paper?

If you can answer no to all of the above, write 'I declare that I have no competing interests' below. If your reply is yes to any, please give details below.

I declare that I have no competing interests

I agree to the open peer review policy of the journal. I understand that my name will be included on my report to the authors and, if the manuscript is accepted for publication, my named report including any attachments I upload will be posted on the website along with the authors' responses. I agree for my report to be made available under an Open Access Creative Commons CC-BY license (http://creativecommons.org/licenses/by/4.0/). I understand that any comments which I do not wish to be included in my named report can be included as confidential comments to the editors, which will not be published.

I agree to the open peer review policy of the journal
